# Supplementary material for: No-Go Decay mRNA cleavage in the ribosome exit tunnel produces 5′-OH ends phosphorylated by Trl1
Source: Nat Commun. 2020 Jan 8;11:122. doi: 10.1038/s41467-019-13991-9 (PMC6949252; doi:10.1038/s41467-019-13991-9)
Supplement: Supplementary file 1 — Supplementary Information [file 41467_2019_13991_MOESM1_ESM.pdf]

Supplementary Information

No-Go Decay mRNA cleavage in the ribosome exit tunnel produces 5’-OH ends phosphorylated by Trl1

Navickas et al.

Supplementary Notes

Supplementary Note 1 Ribosome protection of 3’-NGD RNA fragments from Xrn1 activity. We first focused on the fate of the major B1 (47 nts) and B5 (77 nts) RNA species detected in *dom34* cell extracts, which likely correspond to RNAs protected from Xrn1 digestion *in vivo* by trisomes and disomes, respectively. We showed that Xrn1 treatment of RNA in *dom34* cell extracts had no impact on B5 and B1 RNAs *in vitro,* suggesting that these RNA species are indeed protected by ribosomes (Supplementary Fig. 1b). Interestingly, the persistence of the 71-nt B4 RNA after Xrn1 treatment suggests that this RNA may also be protected by up to three ribosomes in the *dom34* background (Supplementary Fig. 1b).

We also added purified Xrn1 to cell extracts of the *dom34/xrn1* strain *in vitro* and showed that it can efficiently recapitulate the production of the B1 species observed in Xrn1-containing cells *in vivo*. The appearance of the B1 RNA was inversely correlated to the amount of B4, B3 and B2 RNAs remaining, suggesting that these three species have unprotected 5’-protruding RNA extremities *in vivo,* due to the absence of the 5’-3’ exoribonuclease Xrn1 (Supplementary Fig. 1b). The B5 RNA appears to be also generated at the expense of some larger species by Xrn1 treatment *in vitro*, consistent with the presence of trisomes on this species in the *dom34/xrn1* cell extracts (Supplementary Fig. 1b). Based on these experiments, we propose that the B1 (47 nts) and B5 (77 nts) species correspond to Xrn1-trimmed RNAs protected by two and three ribosomes, respectively[1] and that at least some portion of the 71 nt B4 RNA is also protected from Xrn1 by three ribosomes.

Supplementary Note 2 Ribosome protection of 3’-NGD RNA fragments from RNase I activity. To validate the presence and number of ribosomes on 3’-NGD RNAs by a third method, and particularly the presence of trisomes on the 77-nt B5 and 71-nt B4 species, we also performed RNase I protection assays on cell extracts of *dom34* and *dom34 /xrn1* strains. We hypothesized that B5 and B4 RNAs protected from RNase I by three ribosomes should be detectable with both probes prA and prD (Supplementary Fig. 1c). The presence of two or three ribosomes on the major RNA species B1, B4 and B5 in *dom34* cells (deduced from primer extension experiments, ribosome association and Xrn1 treatment *in vitro*) would preferentially conduct RNase I to cleave at three major sites, Cut1, 2 and 3 in Supplementary Fig. 1c. After RNase I treatment (Supplementary Fig. 1d, e), the accumulation of RNase I protected RNAs of similar size to B5 is consistent with the hypothesis that this RNA is covered by trisomes in *dom34* mutant extracts (Fig. 1f). It is known that RNase I and Xrn1 cleave about 15 nts[2] and 17 nts[1] upstream of the first nt of the ribosomal A-site, respectively. We thus expected 5’-end of the B5 RNA to be 1-2 nts shorter than the Xrn1 product after RNase I treatment, if we had accurately calculated the positions of ribosomes on this RNA. Indeed, primer extension experiments confirmed that ribosomes protect a 75-nt species from RNase I in the *dom34* background (Supplementary Fig. 1f). The equivalent of the B1 RNA was 46 nt in size after RNase I treatment (Supplementary Fig. 1g). B4 RNA was not detected using probe prA (Supplementary Fig. 1d, h). We wondered whether RNase I cleaved preferentially at Cut3 (Supplementary Fig. 1c), thus preventing the detection of B4 using probe prA. We probed the membrane in Supplementary Fig. 1h with prA (Fig. 1i), and two distinct RNA species were detected corresponding to B5 and B4 processed by RNase I at Cut3, and inefficiently cleaved at the Cut2 site (Supplementary Fig. 1c). We thus propose that the 5’- extremities of B4 RNA are also protected by ribosomes (at least two ribosomes

limiting RNase I attack at Cut2). We conducted the same experiment on the B4 RNA from *xrn1/dom34* cell extracts. These RNAs were sensitive to Xrn1 treatment *in vitro* (Supplementary Fig. 1b), and using probe prD to detect the B4 RNA specifically, we observed that these RNAs were also protected from RNase I to a similar extent as B4 in *dom34* cell extracts (Supplementary Fig. 1i). Thus, whether two or three ribosomes dwell on the 71-nt B4 RNA in *xrn1/dom34* mutant cell extracts is not completely clear as this species is sensitive to Xrn1 *in vitro* (*i.e.* have 5'-ribosome-free extensions that can be pared down to B1 by Xrn1 digestions) (Supplementary Fig. 1b), which would be consistent with protection by two ribosomes, but it is resistant to RNase I (Supplementary Fig. 1i), which is more consistent with three.

Supplementary Fig. 1

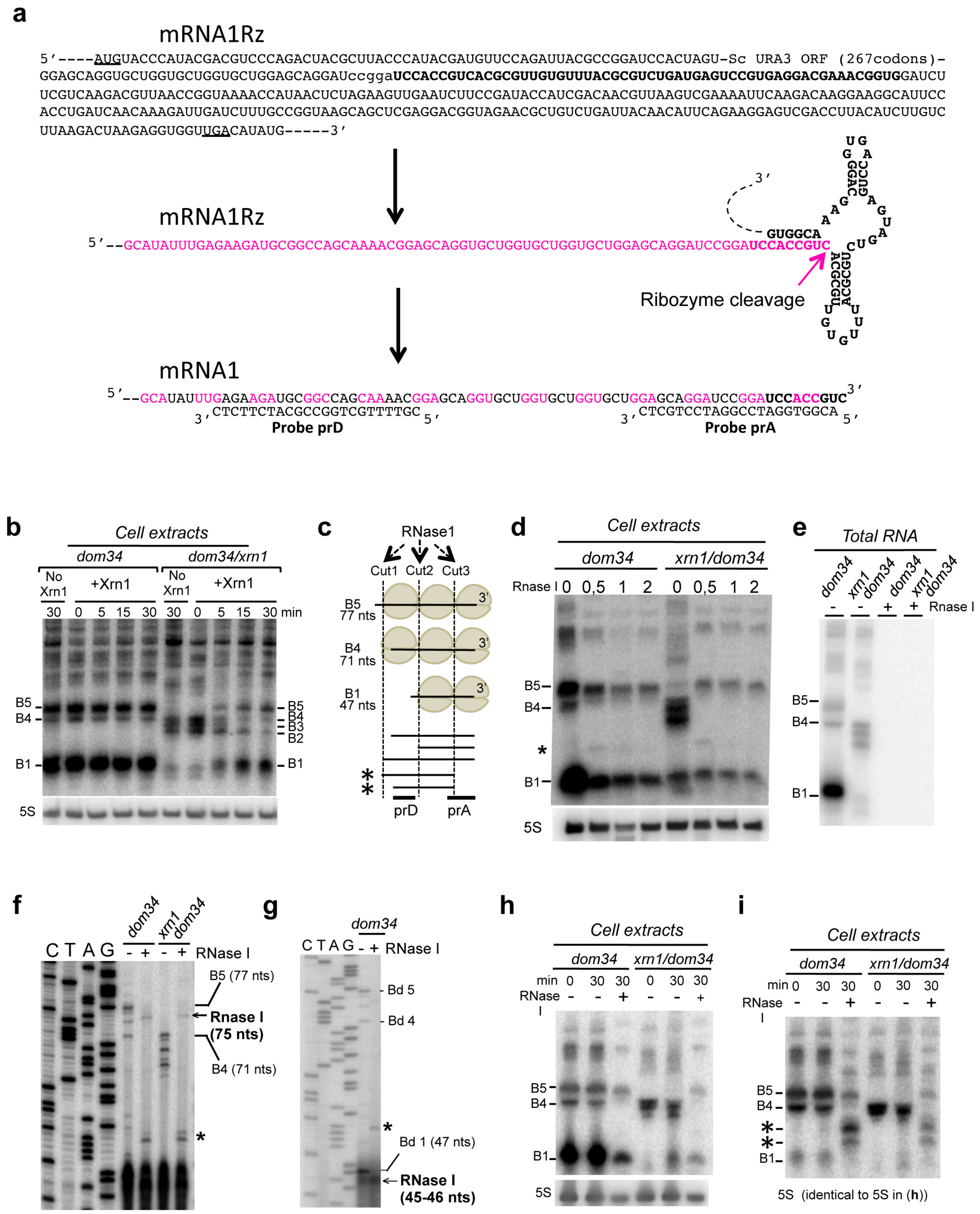

Supplementary Fig. 1 3'-NGD RNA fragment analysis, related to Fig. 1. **a** Sequence of mRNA1Rz. The translational start (AUG) and stop codon (UGA) are underlined. The ribozyme sequence is shown in bold. The magenta arrow indicates the ribozyme cleavage site. mRNA1 is the truncated stop-less codon mRNA after ribozyme cleavage. Probes prA and prD are indicated. **b** Xrn1 treatment *in vitro* of cell extracts (*i.e*. mRNAs in presence of ribosomes) from *dom34* or mutant cells, followed by RNA extraction and northern blot using probe prA.

Sizes in nts are deduced from experiments shown in (Fig. 1c-d). **c** Schematic view of ribosomes covering RNA species B1, B4 and B5 observed in *dom34* mutant cells. Cut1, Cut2 and Cut3 represent potential RNase I cleavage sites. Probes prA and prD used in northern blots analysis shown in (**h**) and (**i**) are indicated. 5'-extremities of B5 and B4 RNAs potentially protected by two ribosomes and detected by prD are indicated by asterisks. **d** RNase I treatment *in vitro* of cell extracts (*i.e.* mRNAs in presence of ribosomes) from *dom34* or *dom34/xrn1* mutant cells followed northern blot using probe prA. The 5S rRNA served as a loading control. 0.5, 1, or 2 μl of RNase I were used (100 units/μl). **e** Similar RNase I treatment analysis that in (**d**) but on extracted RNAs (*i.e.* mRNAs in absence of ribosomes). **f-g** Primer extension experiments using probe prA to determine the 5'-end of RNAs after RNase I treatment of *dom34* and *xnr1/dom34* cell extracts as performed in (**d**). The band indicated by an asterisk is lost at a higher concentration of RNase I as shown in (**d**). **h** RNase I treatment of cell extracts *in vitro*, analysed as in (**b**). **i** The same membrane in (**h**) has been probed with prD. Source data are provided as a Source Data file.

Supplementary Fig. 2

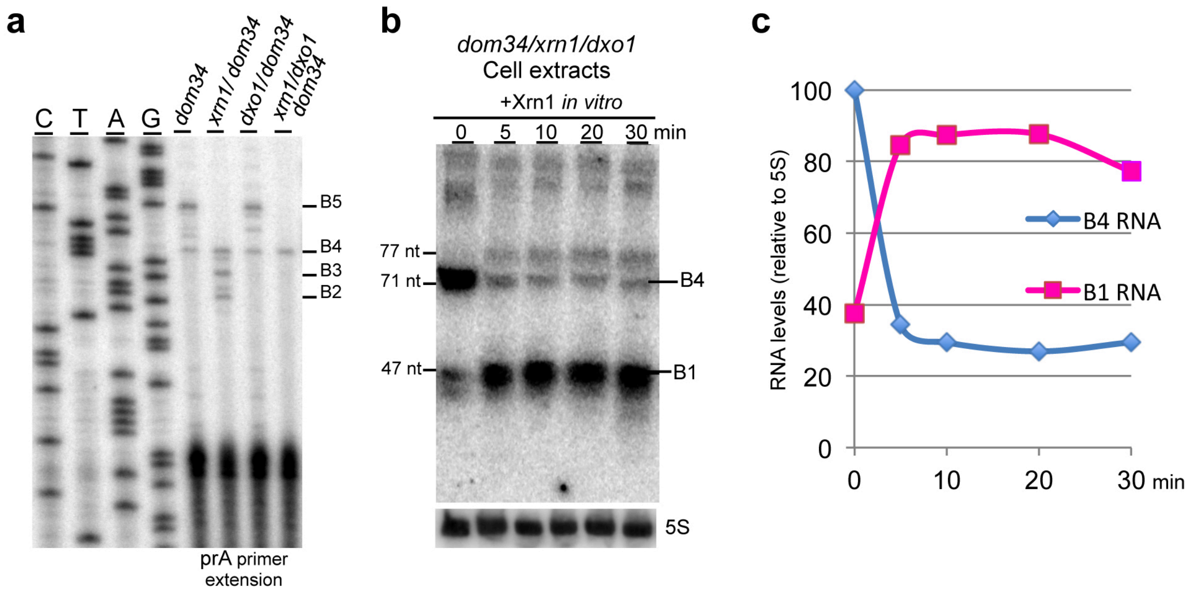

Supplementary Fig. 2 Dxo1 produces heterogeneity of 3'-NGD RNA fragments in Xrn1 deficient cells, related to Fig. 2. **a** Primer extension experiments using probe prA for determining the 5'-end of B1, B2, B3, B4 and B5 3'-NGD RNAs in indicated strains. **b** Cell extracts from *xrn1/dxo1/dom34* cells are digested by Xrn1 *in vitro*. 8% PAGE followed by northern blotting analysis using probe prA. **c** Quantification (a mean of two independent experiments) of RNAs shown in (**b**). B1 RNA (as the 47 nt species) and B4 RNA (as the 71 nt species) are indicated in amount relative to the 5S rRNA. Source data are provided as a Source Data file.

Supplementary Fig. 3

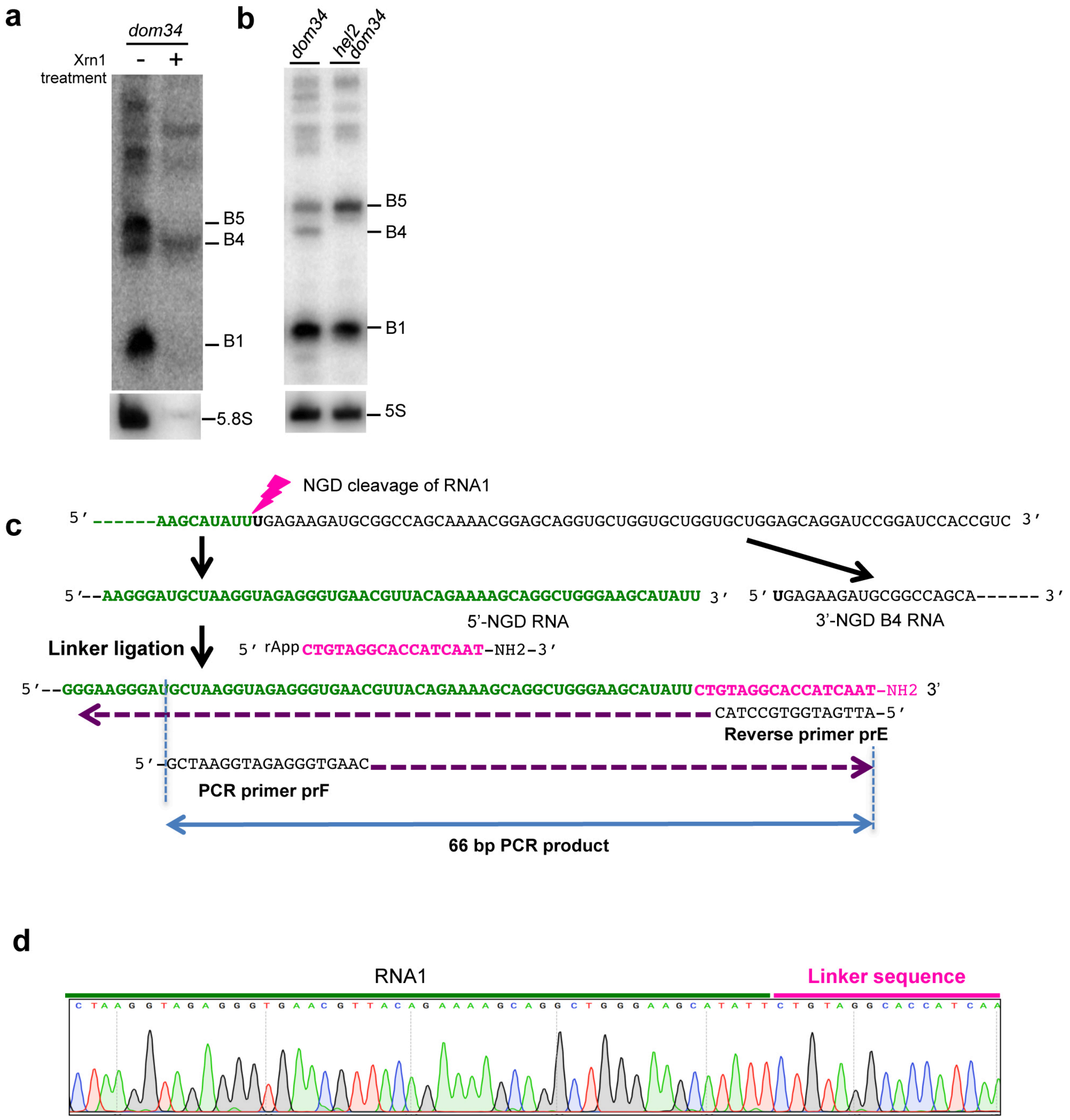

Supplementary Fig. 3 Characterization of B4 RNAs and 3'-RNA ligase mediated RACE, related to Fig. 3. **a** Xrn1 digestion of total RNA extracts from *dom34* mutant cells in Xrn1 buffer *in vitro*. 8% PAGE followed by northern blot analysis using probe prA. The 5.8S rRNA served as a positive control of Xrn1 treatment. **b** B4 RNA production is not detected in *hel2*/*dom34* mutant cells. 8% PAGE followed by northern blotting analysis using probe prA. **c** mRNA1 before and after the endonucleolytic cleavage (represented by the lightning flash) producing the 3'-NGD B4 RNA, and the resulting 5'-NGD RNA. The expected 3'-extremity is shown ligated to the universal miRNA linker (NEB). Sequence of reverse primer prE and PCR primer prF are indicated. A PCR product of 66bp is expected. **d** Chromatogram representing sequences obtained from 3'-RACE experiments performed on total RNA from *ski2* and *ski2*/*dom34* mutant cells. Source data are provided as a Source Data file.

Supplementary Fig. 4

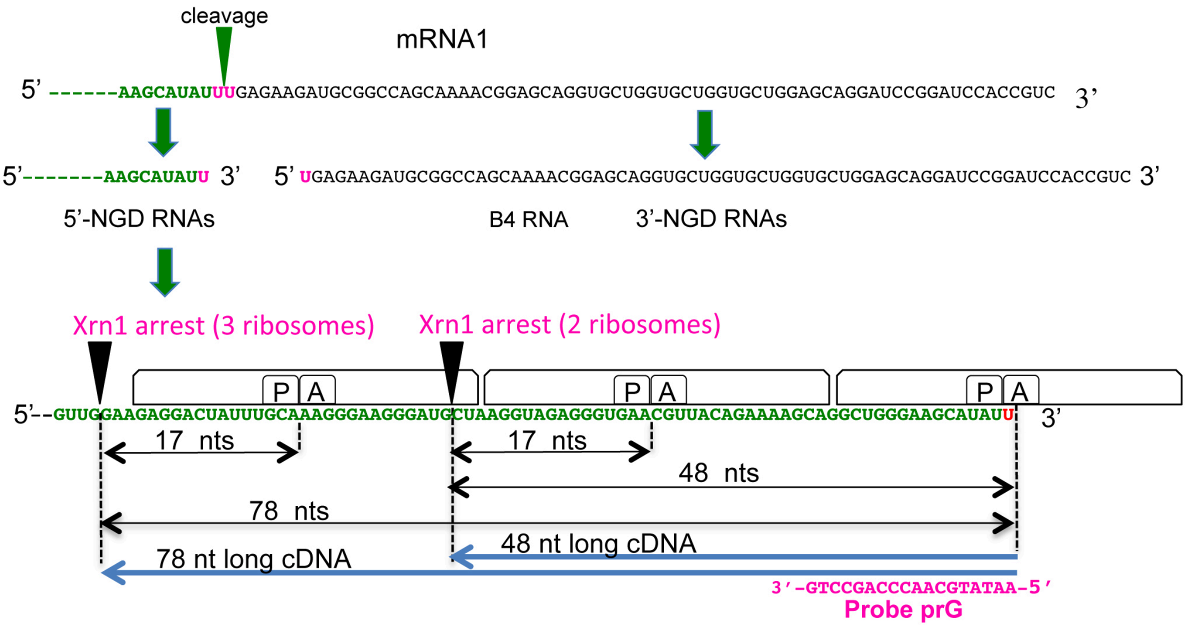

Supplementary Fig. 4 Analysis of the fate of 5'-NGD RNA, related to Fig. 4. Schematic model of mRNA1 before and after the endonucleolytic cleavage producing B4 RNA. The 5'-NGD resulting RNA is shown covered by ribosomes and is shown processed by Xrn1 in 48- and 78-nt RNAs when covered by two and three ribosomes, respectively. Xrn1 arrests occur 17 nts upstream of ribosomal A-site first residues[1]. Using probe prG in primer extension experiments, 48- and 78-nt cDNA products are expected.

Supplementary Fig. 5

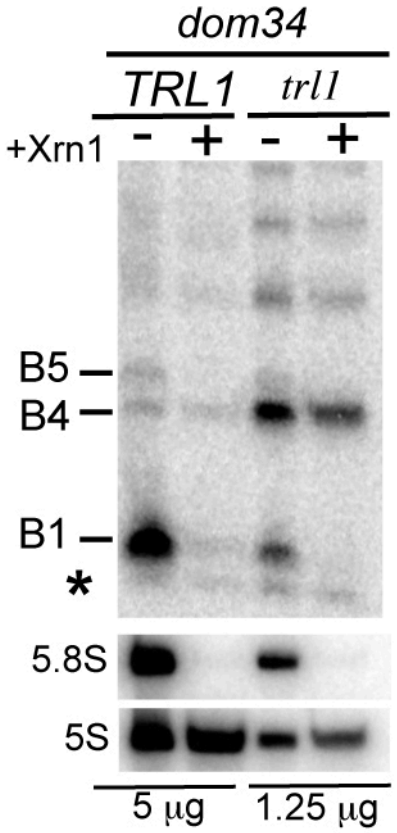

Supplementary Fig. 5 Related to Fig. 5, endonucleolytically cleaved 5'-OH RNAs are phosphorylated by Trl1. Xrn1 digestion of total RNA extracts from *TRL1*/*dom34* and *trl1*/*dom34* mutants in Xrn1 buffer. 5 μg and 1.25 μg of total RNA, from *TRL1* and *trl1* cell extracts respectively, were treated. A minor band detected in *TRL1* and *trl1* cells is indicated by an asterisk. The 5S rRNA served as a loading control. The 5.8S rRNA served as a positive control of Xrn1 treatment. Source data are provided as a Source Data file.

Supplementary Fig. 6

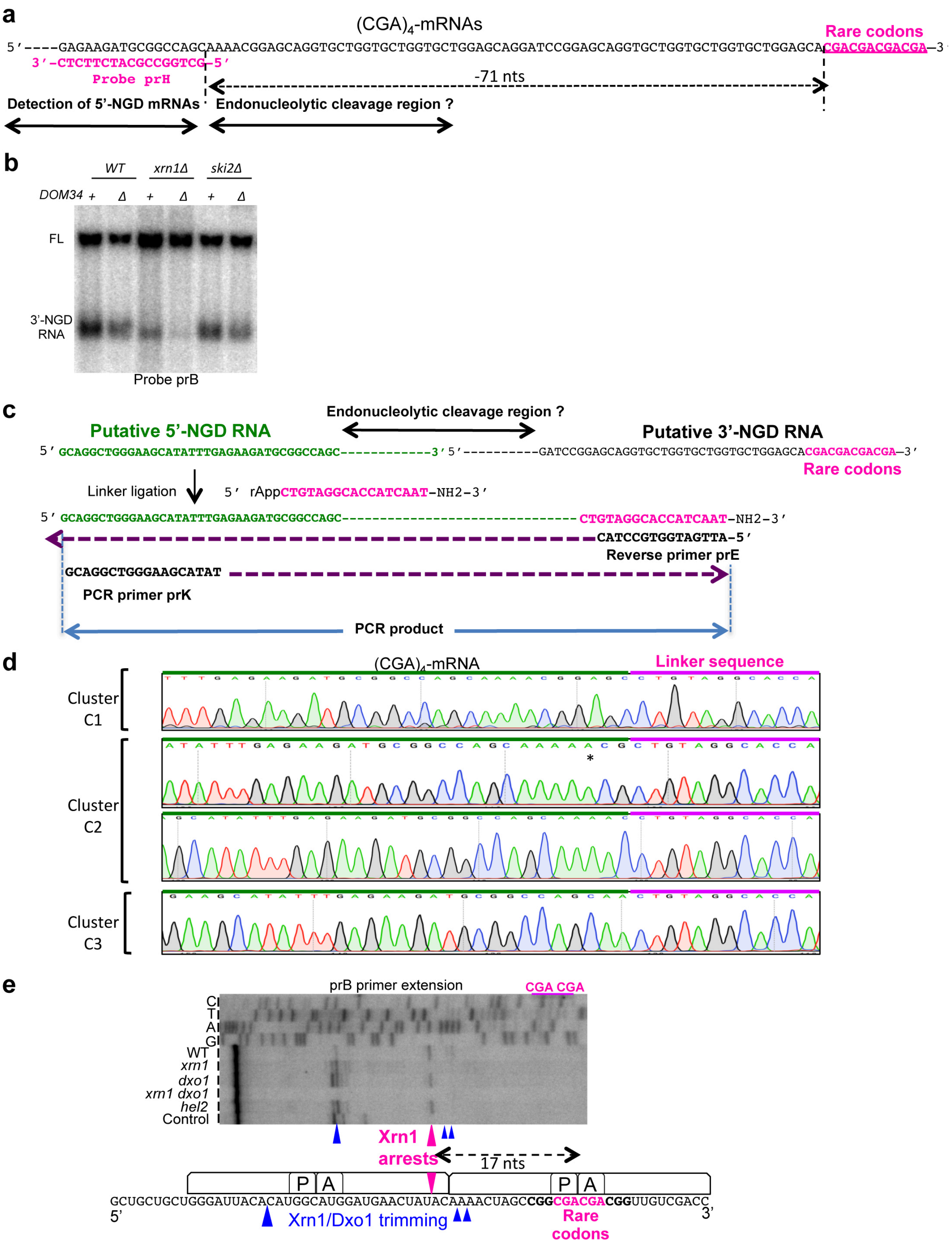

Supplementary Fig. 6 Analysis of (CGA)$_4$-mRNAs, related to Fig. 6. **a** Partial sequence of (CGA)$_4$-mRNA showing region upstream the four CGA rare codons. Positioning of probes prH is indicated. **b** 1.4% agarose gel followed by northern blotting analysis using probe prB showing steady state levels of RNAs in *dom34* and other indicated mutant strains. Full length

$(CGA)_4$-mRNA is noted FL, and the 3’-NGD RNAs are indicated. **c** 3’- RACE. The region of potential endonucleolytic cleavage, the 3’- and 5’-NGD RNAs are indicated. The putative 3’-extremity is shown ligated to the universal miRNA linker (NEB). Sequence of reverse primer prG and PCR primer prK are indicated. **d** Chromatogram representing sequences obtained from 3’-RACE experiments performed on total RNA from *ski2* mutant cells and the three cleavage clusters C1, C2 and C3. The asterisk indicates one nucleotide A mismatch found in sequences. **e** Primer extension experiments using probe prB to determine the 5’-end of the mRNA containing two contiguous CGA rare codons as described previously[3]. A schematic view of the ribosome positioning on this mRNA is shown below and Xrn1-specific arrest is indicated by a magenta arrowhead. Arrests dependent on Xrn1/Dxo1 activities are also indicated by blue arrowheads. Source data are provided as a Source Data file.

Supplementary Methods

Yeast Media. Strains were grown in YPD medium or in synthetic minimum media (SD)[4]. Minimal media was completed for auxotrophy, leucine, histidine and/or uracil were omitted to keep selection for plasmids when necessary. 200 μg/ml G418 Sulfate (Geniticin, American Bioanalytical), 100 μ g/ml Hygromycin B (Sigma-Aldrich) and 100 μ g/ml ClonNat (Werner Bioagents) were added in YPD media plates to select for KanMX4, HphMX4 and NatMX6 respectively.

Strains used in this study. Mutant strains were generated by the one-step gene replacement using PCR fragment of the NatMX6 cassette amplified from plasmid pFA6a-natMX6[5],with HphMX4 containing cassette amplified from pAG32[6] or by the KanMX6 cassette amplified by PCR from plasmid pFA6a-kanMX6 respectively. Correct integration was confirmed by PCR with primers. See Supplementary Table 1 for strains, and Supplementary Table 2 for used primers.

Plasmids used in this study. Yeast plasmids used in this study were constructed using standard molecular biology procedures. To construct pLB138 (mRNA1RZ) and pLB127 ($(CGA)_4$-mRNA), p415ADH1[7] was first digested by SpeI-XhoI. DNA fragments containing URA3 were amplified by PCR from pRS316[8] using primers olb592-olb593 and digested by SpeI-BamHI. In parallel, oligonucleotides olb-ins1-f and olb-ins1-r were annealed. All DNA fragments were ligated to build pADH1-URA3. pADH1-URA3 was then digested by BspEI-NdeI. Genomic DNA was amplified using primers olb594-olb596 and digested by BamHI-NdeI in order to insert an additional ORF (ORF2) in the 3'-region of *URA3*. In parallel, in order to insert a ribozyme sequence (Rz) just downstream URA3 sequence, oligonucleotides olb625 and olb626 were annealed and all DNA fragments were ligated to form pADH1-URA3-Rz-ORF2. To insert 4 CGA codons, oligonucleotides olb640 and olb641 were annealed and all DNA fragments were ligated to form pADH1-URA3-$(CGA)_4$-ORF2. Additionally, oligonucleotides olb-2HA-f and olb-2HA-r were annealed and cloned into pADH1-URA3-Rz-ORF2 or pADH1-URA3-(CGA)4-ORF2 (XbaI-SpeI digestion). The resulting plasmids p415ADH1-2HA-URA3-Rz-ORF2 and p415ADH1-2HA-URA3-$(CGA)_4$-ORF2 were named p138 and p127 respectively. The resulting ORF sequence of the mRNA with 3'-Rz insertion is shown in Supplementary Fig. 1a. Plasmids $pDxo1_{WT}$ and $pDxo1_{mut}$ used for the expression *in vivo* of WT Dxo1-Flag or of a catalytic mutant of Dxo1 (E260A D262A) were both created using synthetized DNAs (Genecust) cloned in SalI- XbaI sites of pRS313 (synthetized DNA sequences in Table S4). Thermocompetent NEB 10-beta *E. coli* (NEB) were used for cloning; all the plasmids were verified by sequencing (Eurofins Genomics).

Supplementary Tables

Supplementary Table 1. Strains used in this study

Supplementary Table 2. Oligonucleotides used in this study

Supplementary Table 3. Synthetized DNA DXO1 sequences for plasmid constructions

Supplementary Table 4. Plasmids used in this study

Supplementary Table 1

**Name**	**alias**	**genotype**	**ref.**	
BY4741	WT	*MATa his3Δ1 leu2Δ0 met15Δ0 ura3Δ0*	euroscarf	
Y05329	*dom34*	*MATa his3Δ1 leu2Δ0 met15Δ0 ura3Δ0 dom34::kanMX4*	euroscarf	
BY11756	*met22*	*MATa his3Δ1 leu2Δ0 lys2Δ0 ura3Δ0 met22::kanMX4*	euroscarf	
Y04540	*xrn1*	*MATa his3Δ1 leu2Δ0 met15Δ0 ura3Δ0 xrn1::kanMX4*	euroscarf	
Y05307	*ski2*	*MATa his3Δ1 leu2Δ0 met15Δ0 ura3Δ0 ski2::kanMX4*	euroscarf	
YLB152	*dxo1*	*MATa his3Δ1 leu2Δ0 met15Δ0 ura3Δ0 dxo1::kanMX4*	euroscarf	
YLB177	*met22 xrn1*	*MATa his3Δ1 leu2Δ0 lys2Δ0 ura3Δ0 met22::kanMX4 xrn1::hphMX4*	this study	derived from BY11756
YLB082	*ski2 dom34*	*MATa his3Δ1 leu2Δ0 lys2Δ0 ura3Δ0 ski2::kanMX4 dom34::natMX6*	this study	derived from Y05307
YLB083	*xrn1 dom34*	*MATa his3Δ1 leu2Δ0 lys2Δ0 ura3Δ0 xrn1::kanMX4 dom34::natMX6*	this study	derived from Y04540
YLB084	*met22 dom34*	*MATa his3Δ1 leu2Δ0 lys2Δ0 ura3Δ0 met22::kanMX4 dom34::natMX6*	this study	derived from BY11756
YLB178	*dxo1 dom34*	*MATa his3Δ1 leu2Δ0 met15Δ0 ura3Δ0 dxo1::kanMX4 dom34::natMX6*	this study	derived from YLB152
YLB179	*xrn1 dxo1 dom34*	*MATa his3Δ1 leu2Δ0 met15Δ0 ura3Δ0 dxo1::kanMX4 xrn1::hphMX4 dom34::natMX6*	this study	derived from YLB178
YLB176	*xrn1 met22 dom34*	*MATa his3Δ1 leu2Δ0 lys2Δ0 ura3Δ0 met22::kanMX4 dom34::natMX6 xrn1::hphMX4*	this study	derived from YLB177
YLB302	*ltn1 dom34*	*MATa his3Δ1 leu2Δ0 met15Δ0 ura3Δ0 ltn1::kanMX4 dom34::natMX6*	this study	derived from Y04540
YLB303	*hel2 dom34*	*MATa his3Δ1 leu2Δ0 met15Δ0 ura3Δ0 hel2::kanMX4 dom34::natMX6*	this study	derived from Y04540
YJC432	dcp2	*MATa his3Δ1 leu2Δ0 met15Δ0 ura3Δ0 dcp2::neoMX*	Jeff Coller	9
YLB156	*dcp2 dom34*	*MATa his3Δ1 leu2Δ0 met15Δ0 ura3Δ0 dcp2::neoMX dom34::natMX6*	this study	
YJH682	*WT*	*MATa leu2-3 112 trp1-1 can1-100 ura3-1 ade2-1 his3-11,15*	Jay Hesselberth	10,11
YJH835	*trl1*	*MATa leu2-3 112 trp1-1 can1-100 ura3-1 ade2-1 his3-11,15 trl1::KanMX (pAG424-10x-tRNA)*	Jay Hesselberth	10,11
YLB313	*dom34*	*MATa leu2-3 112 trp1-1 can1-100 ura3-1 ade2-1 his3-11,15 dom34::NatMX*	this study	
YLB317	*trl1 dom34*	*MATa leu2-3 112 trp1-1 can1-100 ura3-1 ade2-1 his3-11,15 trl1::KanMX (pAG424-10x-tRNA) dom34::NatMX*	this study	

Supplementary Table 2

Name	alias	Oligo used for	Sequence 5' to 3'
olb495		DOM34 deletion by natMX6	CATTCGTTGCTGCATCGTTGTCATTTTGTTCAATTATCGCATTCCTATCATAGCAAAAATCG GATCCCCGGGTTAATTAA
olb496		DOM34 deletion by natMX6	CGATTTATTATAGGGTTGCAAATTTTATGTGTACATTACTTTTTTCTTACATAGTAAATCGA ATTCGAGCTCGTTTAAAC
olb497		DOM34 deletion verification	GCGTCATCTTCTAACACCG
olb498		DOM34 deletion verification	GTGAACAGGTTCAGACAACTTCAAAGC
olb372		XRN1 deletion by hphMX4	GTTTATTTTCTAAAGGATACTGTCTTCTTCCGTACTTATAATCGGGTTCACGATCTGTTTAG CTTGCCTTG
olb373		XRN1 deletion by hphMX4	CAATCCCCATTTGTTATAAGCTTTTTCTTAACAAGATCAACGATTAAATACCTCGTTTTCGA CACTGGATG
olb379		XRN1 deletion verification	CTATTCTCACGATTAATGGTC
olb380		XRN1 deletion verification	ATGGGAGACGTGCAAAAGC
olb561		SKI2 deletion verification	GGAACGGATAGAGGTTTGAAAAAGG
olb562		SKI2 deletion verification	GCTTTGGTTCATCGGTGCTC
olb978		DXO1 deletion verification	CCGCAATTTTCGCAGCATG
olb979		DXO1 deletion verification	GGTTTACGCATTGCTTTTCATAG
olb592		Construction of pLB138 (URA3 insert /URA3_SpeI)	GACATACTAGTATGTCGAAAGCTACATATAAGGAACG
olb593		Construction of pLB138 (URA3 insert/URA3_BamHI)	GACATGGATCCTGCTCCAGCACCAGCACCAGCACCTGCTCCGTTTTGCTGGCCGCATC
olb Ins1-f		insert in 3' of URA3 BglII-NdeI sites	GATCCCCAGTAGATCTTCGTGGGTGACATATGCCCGGGCTCGAGTGATAAG
olb Ins1-r		insert in 3' of URA3 BglII-NdeI sites	TCGACTTATCACTCGAGCCCGGGCATATGTCACCCACGAAGATCTACTGGG
olb594		Construction of pLB138 (downstream ORF of URA3_BamH1I)	GACATGGATCCGGAGCAGGTGCTGGTGCTGGTGCTGGAGCAATGCAGATCTTCGTCAAGACG
olb596		Construction of pLB138 (downstream ORF of URA_Nde1I)	GATACCATATGTCAACCACCTCTTAGTCTTAAGAC
olb625		Construction of pLB138 (RZ insertion)	CCGGATCCACCGTCACGCGTTGTGTTTACGCGTCTGATGAGTCCGTGAGGACGAAACGGTG
olb626		Construction of pLB138 (RZ insertion)	GATCCACCGTTTCGTCCTCACGGACTCATCAGACGCGTAAACACAACGCGTGACGGTGGAT
olb640		Construction of pLB127 (CGA4 insertion)	CCGGAGCAGGTGCTGGTGCTGGTGCTGGAGCACGACGACGACGAATGCA
olb641		Construction of pLB1278 (CGA4 insertion)	GATCTGCATTCGTCGTCGTCGTGCTCCAGCACCAGCACCAGCACCTGCT
olb 2HA-f		insert 2HA in XbaI-SpeI	CTAGAGGATCTATGTACCCATACGACGTCCCAGACTACGCTTACCCATACGATGTTCCAGAT TACGCCGGATCCA
olb-2HA-r		insert 2HA in XbaI-SpeI	CTAGTGGATCCGGCGTAATCTGGAACATCGTATGGGTAAGCGTAGTCTGGGACGTCGTATGG GTACATAGATCCT
olb138-rev		Construction of pLB138	CTTCCCAGCCTGCTTTTC
olb138-22f		Construction of pLB138-22 (with olb138-rev)	GCTGGGAAGAAGCAUAUUUGAGAAGAUGGGCCAGCAAAACGGAGC
olb138-23f		Construction of pLB138-23 (with olb138-rev)	GCTGGGAAGAAAGCAUAUUCUAGAAGAUGGCCAGCAAAACGGAGC
olb138-24f		Construction of pLB138-24 (with olb138-rev)	GCTGGGAAGAGCAUAUAGAUUUUUUUGCGGCCAGCAAAACGGAGC
olb917	prA	probe of mRNA1	ACGGTGGATCCGGATCCTGCTC
olb622	prB	probe of mRNA1	CCGGGCATATGTCAACCACCTC
olb621	prC	probe of mRNA1	GGCGTAATCTGGAACATCGTATG
olb994	prD	probe of mRNA1	CGTTTTGCTGGCCGCATCTTCTC
olb937	prE	complementary sequence to universal miRNA cloning linker	ATTGATGGTGCCTACAG
olb1101	prF	probe of mRNA1 , PCR pirmer for 3' RNA ligae mediated RACE	GCTAAGGTAGAGGGTGAAC
olb1058	prG	probe of mRNA1 mRNA	AATATGCTTCCCAGCCTG
olb1117	prH	probe of mRNA-CGA4	GCTGGCCGCATCTTCTC
olb933	prJ	for primer extension on mRNA-CGA4	GTTGTCGATGGTATCGGAAGATTC
olb1104	prK	probe of RNA-CGA4 , PCR pirmer for 3' RNA ligase mediated RACE	GCAGGCTGGGAAGCATAT

Supplementary Table 2 (continued)

Name	alias	Oligo used for	Sequence 5' to 3'
olb378	scR1	detection of scR1 mRNA	GTCTAGCCGCGAGGAAGG
olb25S	25S	detection of 25S rRNA	ATCCGCTAAGGAGTGTGTAACAACTCACC
olb18S	18S	detection of 18S rRNA	AGCCATTCGCAGTTTCACTG
olb959	5S	detection of 5S rRNA	CTACTCGGTCAGGCTCTTAC
olb1112	5.8S	detection of 5.8S rRNA	CCAAGAGATCCGTTGTTGAAAG

Supplementary Table 3

Sequences

Synthetized DNA sequences

Sc WT Dxo1 used forconstruction of pDxo1wt

GTCGACGAGACACTAAGCTGCTTTTTGATCTGTGCTGAGCTGAGTGTCCTACCATATGATCACTCTTGTATCGTCTACTT
ATAGAGGTTGTTAGTACCAACCTGCAAATACAATTGTTGGACCATACTACCACAAAAGGTTCTTTTCGTTGGTGTATCCT
CACAAATAATGATTATTCCACCAAAATCACCAAATTATTGTGTCAAACTAACTGGGATTACCCCACTATTGATTTTTCCA
GTTTTTCCGCAATTTTCGCAGCATGTTTTACTTTTATAATGGCATTGTTGCATTACAGCGCCAGTTGTAGGTATCGATGA
TTTTATGTCAAAGTTTTCTTTCTAGCCAAACTCAGTTATGTCAACTGAACAAGATGCTGTTCTTGGATTGGCCAAAGATT
TAGAAGGTATAAATTTGCTTACTGTGCCCAATCTCGAGAGAGGACACCAAAGTAAATTATGCAAAGAGAAAACTACTTCT
GATTCATCTTCGTCAAGGAAGCCTTCACAACAGAGAGACAATTATAGAAAGAGACGTCCGAAACTTATATGTATCCCATA
TACGTCTTTTCTGCATACTGGTATGCACAATTTTTTGACGAAACCACCAAGAGATATATTTCATGAAAGTAAAGAAGTAG
CTCTGTTTACCAATGGCCGGGCTTATACAATCCTACGCAAAGACCTTATACCAAATTTGAAAGAAAGTATTGCTGAATTG
TATGAAAGCTCGCTTCTTGAGGCAAAAAAGCGGAAAGTCCCGTATTTAGGCCATGACTTATTTGCTAATATTGATGAGTT
CGTTCCCATGACAATATCCGAATTAGATAGTGTATCACCGTGTTTTTCATACATTGAGAACTGGATACTAGATAATCCTG
GTAAGGATTTTAAGATCGGCAAGAAATTTACTGTTGTAACCACAAGACATCATATCGTAGATTTGACTATGCATCTCTTT
AACAGGCGAAATAGACAAACGTCACTAATTGTAACTTATATGGGGGCGGGCCTTCTTTCATTTTGCAGAAATGTAAAAAA
AGATTCTCAAATGTCCAAAGAGGGCATTTATTCAAATGATCCAAATATGAAGAAAATTTGCTATTCAGGATTTGAATTTG
AAAATTGGGTAACCGAAAATTCCAAAGTCGCTGATTTAACTGGCTCTAAATGTCCTATTTTTTCTCTTGTAGAGAGTAAA
CTTTCAGAAGAAATTGGTCTTTTAATTCGCTGCGAAATGGATGCATTCAATCCTGTTTCGGAGACAAACACAGAACTAAA
GTGTTTTGCCCCATTATCAATGCACAATTCCAATCATAGGAGAAAACTTCTGAAAACGTGGGTACAGACGGGTTTATTAC
CGAACTCAGATATCATGATAGGTTTGAGGGACAGTCATAGCGGTCAATTACTAGACATTCAATGGTACTCAAGGGACTTA
TTATGTAAGAAATTCAATCACCCAGGTCTACCTACAAATAAAAAGGAACTTAACTATAATGCCCAAATTGCGGTAGAATG
GTGTCATTATTGTATTGAAGCAATTTGTAAGCTGGTGGAGGCAAATATCTCTGACTATAGCAGTACAAAACCAGAATCAT
TTGAAATCGGTATAGATACTAACAACGCCATCGTCATCACTAAACTTAAGACTACTCCAAGAAACGTAGAATTATTTGGA
ATGggatccggtgctggtgctggtgctggagcagattataaagatgacgatgacaaggactacaaggacgatgatgacaa
aggatccTAGTAAAGACGTGTATAATATATAATACTTTTCCGAGAAATATTTCATTTTCATTTTCGTAAAGTTGTTAACT
ACGCTAAATATTAGTACTTTTCTTAAATTTATATGGGGAGCCCTTTTTTTCTATGAAAAGCAATGCGTAAACCAAATAAG
CAGAATTTTGTAATAGATGAGCAACAATACTGAGAAGGTGATAACTATAAATTTATGTGGGTAGTACGATACCAGAATAC
ATTGGAAGATGGCTCTATTAGCTTTATATCATGTTGTCTTCAGGCCTTCAAAACTTATAGTATAGGTAGATCATCTAGA

Sc Dxo1 mutant used for construction of pDxo1mut (E260A/D262A)

GTCGACGAGACACTAAGCTGCTTTTTGATCTGTGCTGAGCTGAGTGTCCTACCATATGATCACTCTTGTATCGTCTACTT
ATAGAGGTTGTTAGTACCAACCTGCAAATACAATTGTTGGACCATACTACCACAAAAGGTTCTTTTCGTTGGTGTATCCT
CACAAATAATGATTATTCCACCAAAATCACCAAATTATTGTGTCAAACTAACTGGGATTACCCCACTATTGATTTTTCCA
GTTTTTCCGCAATTTTCGCAGCATGTTTTACTTTTATAATGGCATTGTTGCATTACAGCGCCAGTTGTAGGTATCGATGA
TTTTATGTCAAAGTTTTCTTTCTAGCCAAACTCAGTTATGTCAACTGAACAAGATGCTGTTCTTGGATTGGCCAAAGATT
TAGAAGGTATAAATTTGCTTACTGTGCCCAATCTCGAGAGAGGACACCAAAGTAAATTATGCAAAGAGAAAACTACTTCT
GATTCATCTTCGTCAAGGAAGCCTTCACAACAGAGAGACAATTATAGAAAGAGACGTCCGAAACTTATATGTATCCCATA
TACGTCTTTTCTGCATACTGGTATGCACAATTTTTTGACGAAACCACCAAGAGATATATTTCATGAAAGTAAAGAAGTAG
CTCTGTTTACCAATGGCCGGGCTTATACAATCCTACGCAAAGACCTTATACCAAATTTGAAAGAAAGTATTGCTGAATTG
TATGAAAGCTCGCTTCTTGAGGCAAAAAAGCGGAAAGTCCCGTATTTAGGCCATGACTTATTTGCTAATATTGATGAGTT
CGTTCCCATGACAATATCCGAATTAGATAGTGTATCACCGTGTTTTTCATACATTGAGAACTGGATACTAGATAATCCTG
GTAAGGATTTTAAGATCGGCAAGAAATTTACTGTTGTAACCACAAGACATCATATCGTAGATTTGACTATGCATCTCTTT
AACAGGCGAAATAGACAAACGTCACTAATTGTAACTTATATGGGGGCGGGCCTTCTTTCATTTTGCAGAAATGTAAAAAA
AGATTCTCAAATGTCCAAAGAGGGCATTTATTCAAATGATCCAAATATGAAGAAAATTTGCTATTCAGGATTTGAATTTG
AAAATTGGGTAACCGAAAATTCCAAAGTCGCTGATTTAACTGGCTCTAAATGTCCTATTTTTTCTCTTGTAGAGAGTAAA
CTTTCAGAAGAAATTGGTCTTTTAATTCGCTGCGCTATGGCTGCATTCAATCCTGTTTCGGAGACAAACACAGAACTAAA
GTGTTTTGCCCCATTATCAATGCACAATTCCAATCATAGGAGAAAACTTCTGAAAACGTGGGTACAGACGGGTTTATTAC
CGAACTCAGATATCATGATAGGTTTGAGGGACAGTCATAGCGGTCAATTACTAGACATTCAATGGTACTCAAGGGACTTA
TTATGTAAGAAATTCAATCACCCAGGTCTACCTACAAATAAAAAGGAACTTAACTATAATGCCCAAATTGCGGTAGAATG
GTGTCATTATTGTATTGAAGCAATTTGTAAGCTGGTGGAGGCAAATATCTCTGACTATAGCAGTACAAAACCAGAATCAT
TTGAAATCGGTATAGATACTAACAACGCCATCGTCATCACTAAACTTAAGACTACTCCAAGAAACGTAGAATTATTTGGA
ATGggatccggtgctggtgctggtgctggagcagattataaagatgacgatgacaaggactacaaggacgatgatgacaa
aggatccTAGTAAAGACGTGTATAATATATAATACTTTTCCGAGAAATATTTCATTTTCATTTTCGTAAAGTTGTTAACT
ACGCTAAATATTAGTACTTTTCTTAAATTTATATGGGGAGCCCTTTTTTTCTATGAAAAGCAATGCGTAAACCAAATAAG
CAGAATTTTGTAATAGATGAGCAACAATACTGAGAAGGTGATAACTATAAATTTATGTGGGTAGTACGATACCAGAATAC
ATTGGAAGATGGCTCTATTAGCTTTATATCATGTTGTCTTCAGGCCTTCAAAACTTATAGTATAGGTAGATCATCTAGA

Supplementary Table 4

Plasmid	Description	Marker	Reference
p138	expression of RNA1Rz	LEU2	This study
p127	expression of (CGA)4-mRNA	LEU2	This study
p127T	expression of (CGA)2-mRNA	LEU2	This study
pDxo1WT	expression of Dxo1 of S. cerevisiae from pRS313 plasmid derivative	HIS3	This study
pDxo1mut	expression of a catalytic mutant of Dxo1 of S. cerevisiae from pRS313 plasmid derivative	HIS3	This study
pFA6a-natMX6	natMX6 confers resistance to nourseothricin		5
pAG32	hphMX4 confers resistance to hygromycin B		6
pRS316	Centromeric	URA3	8
pRS313	Centromeric	HIS3	8

Supplementary References

1. Pelechano, V., Wei, W. & Steinmetz, L.M. Widespread Co-translational RNA Decay Reveals Ribosome Dynamics. *Cell* **161**, 1400-1412 (2015).

2. Guydosh, N. R. & Green, R. Translation of poly(A) tails leads to precise mRNA cleavage. *RNA* **23**, 749-761 (2017).

3. Tsuboi, T. et al. Dom34:hbs1 plays a general role in quality-control systems by dissociation of a stalled ribosome at the 3' end of aberrant mRNA. *Mol. Cell* **46**, 518-529 (2012).

4. Adams, A., Gottschling, D.E., Kaiser, C.A. & Stearns, T. Methods in yeast genetics. *Cold Spring Harbor Laboratory Press, Cold Spring Harbor*, NY (1997).

5. Hentges, P., Van Driessche, B., Tafforeau, L., Vandenhaute, J. & Carr, A.M. Three novel antibiotic marker cassettes for gene disruption and marker switching in Schizosaccharomyces pombe. *Yeast* **22**, 1013-1019 (2005).

6. Goldstein, A.L. & McCusker, J.H. Three new dominant drug resistance cassettes for gene disruption in Saccharomyces cerevisiae. *Yeast* **15**, 1541-1553 (1999).

7. Mumberg, D., Muller, R. & Funk, M. Yeast vectors for the controlled expression of heterologous proteins in different genetic backgrounds. *Gene* **156**, 119-122 (1995).

8. Sikorski, R.S. & Hieter, P. A system of shuttle vectors and yeast host strains designed for efficient manipulation of DNA in Saccharomyces cerevisiae. *Genetics* ***122***, 19-27 (1989).

9. Hu, W., Sweet, T.J., Chamnongpol, S., Baker, K.E. & J. Coller. Co-translational mRNA decay in Saccharomyces cerevisiae. *Nature* **461**, 225-229 (2009).

10. Cherry, P.D., White, L.K., York, K. & Hesselberth, J.R. Genetic bypass of essential RNA repair enzymes in budding yeast. *RNA* **24**, 313-323 (2018).

11. Cherry, P.D., Peach, S.E. & Hesselberth, J.R. Multiple decay events target HAC1 mRNA during splicing to regulate the unfolded protein response. *eLife* **8,** e42262 (2019).
