# Supplementary material for: No-Go Decay mRNA cleavage in the ribosome exit tunnel produces 5′-OH ends phosphorylated by Trl1
Source: Nat Commun. 2020 Jan 8;11:122. doi: 10.1038/s41467-019-13991-9 (PMC6949252; doi:10.1038/s41467-019-13991-9)
Supplement: Supplementary file 2 — Peer Review File [file 41467_2019_13991_MOESM2_ESM.pdf]

Reviewers' comments:

Reviewer #1 (Remarks to the Author):

No-go decay (NGD) is a quality control mechanism essential for the degradation of aberrant transcript with propensity to cause ribosomal stall. It is well known that the mRNA is endonucleolytically cleaved near the stalled ribosome. However, precise mechanism that links ribosomal stall to the endonucleolytic cleavage has not been elucidated. In the present study, the authors suggest that (i) endonucleolytic cleavage occurs within the mRNA exit tunnel of the third or upstream ribosome numbered from the stalled site, and (ii) after removal of the third ribosome, the 5′-hydroxylated extremity of the 3′-NGD RNA fragment is phosphorylated by the Rlg1/Trl1 kinase, thereby enabling exonucleolytic degradation by the 5′-3′ exonucleases Xrn1 and Dxo1. Overall, the data presented are generally convincing and interesting, however, a few issues still need to be resolved.

(1) All the results presented are based on the measure of the RNA fragments generated. From these results, the authors conclude that the cleavage of the RNA is due to the binding of the ribosome(s). There is no evidence showing that the RNA fragments (i.e., B1, B2, B3, B4, B5 RNA) observed in this study are resulting from the RNA protected by the binding of ribosome(s). The authors should show ribosome(s) actually bind to the RNA as disome, trisome, etc. by using polysome profile analysis. For example, B1 RNA mainly appeared in the dom34 background is fractionated to the disome?

(2) In Figure 2d and Supplementary Fig. 2d, the authors constructed three derivatives of mRNA1 named mRNA 2, 3 and 4. It is helpful for the reader to know the reason why the RNA sequences are designed as they are.

(3) Figure 3a, Figure 5: the authors should provide direct evidence showing that B4 RNA has a hydroxyl group at its 5′-end (e.g., by using (i) phosphorylation of the 5′OH with T4PNK and 32P-ATP or (ii) oligonucleotide linker ligation followed by reverse transcription and PCR analysis (a method described by Peach et al., NAR 2015)).

(4) In Figure 5a, B3 RNA is mainly observed in xrn1/dom34 cells, which appears to be inconsistent with the data in Figure 1-3. The authors should discuss it.

(5) Some of the nomenclatures used are very confusing. For example, the words "B4" and "Bd4" are used for the same 3′NGD RNA, and "RNA1" and "mRNA1" are used for 5′NGD RNA.

(6) The manuscript contains a lot of typos/errors that need to be corrected throughout the manuscript.
Figure 1a legend: "Stop-less codon mRNA" should be "Stop codon-less mRNA".
Figure 1b legend: "Agarose gel followed by"" should be "Agarose gel electrophoresis followed by".
Figure 1b legend: "Probes prA, and prD" should be "Probes prA and prD".
Figure 1h legend: "prA" should be "prD".
Figure 2d legend: the word "RNA2, RNA3 and RNA4" is used in the legend, while "mRNA 2, 3, 4" is used in the main text, which is confusing to the reader.
Figure 4b legend: "prA" should be "prG" and "prG" should be "prA".
Figure 5: "RGL1" should be "RLG1".
Figure 6C does not appear in the main text.
Supplementary Fig. 5: "RGL1" should be "RLG1".
Page 6, line 5 from the bottom: "hypothetised" should be "hypothesized".
Page 11, line 5, "Supplementary Fig. 1e, 2b) should be "Fig. 1e and Supplementary Fig. 2b).
Page 13, line 4: the word "NGDase" must be explained here.
Page 15 line 7: "in vitro and in vitro" should be "in vitro and in vivo".

Reviewer #2 (Remarks to the Author):

In this manuscript, Navickas and colleagues performed a detailed analysis of RNA fragments derived from No-Go decay reporters in mutant yeast strains and propose a model for this process involving endonucleolytic cleavage of the target mRNA in the third ribosome blocked upstream of the stalling site (or ribosome present further 5').

While this study brings some new detailed information, it is unclear whether the proposed mechanism resulting from analysis of RNA decay intermediate accumulating in mutant cells, represents the main No-Go decay pathway. Indeed, most analyses are performed in mutants with inactivated Dom34. While based on bibliographic data authors argue that endonucleotlytic cleavage still occurs in this context, previous analyses have shown that full length mRNA stability is clearly increased in the absence of Dom34 indicating that endonucleolytic mRNA cleavage is blocked. The authors data presented in Figure 1b support further the fact that in the absence of Dom34 endonucleolytic RNA decay is strongly reduced (compare levels of full-length mRNA in the presence and absence of Dom34), leaving the possibility that RNA degradation events occurring in a dom34 mutant context represent minor side pathways. Consistently, previously published data suggested that the initial attack of No-Go decay substrate occurred much closer to the ribosome stall site.
Moreover, some of the data presented are difficult to reconcile with the authors' interpretation. Hence, authors propose that intermediates generated by endonucleolytic cleavage in the absence of Dom34 have a 5'OH group that is phosphorylated by Rgl1 before Xrn1 digestion. If this is indeed the case, it is unclear why the levels of B5 and B1 decay intermediates increase in a rgl1-4 dom34 double mutant compared to a single dom34 mutant (Figure 5a, compare to loading control). Indeed, according to the authors model, those B5 and B1 intermediates are expected to be dependant of Rgl1 activity and thus their levels are expected to decrease in the presence of a less active/inactive Rgl1.

Overall, while this study presents a detailed study of decay intermediates derived from No-Go decay reporters in mutant yeast strains, the relation of these products to events occurring during No-Go decay in wild-type cells remain partly fuzzy. In the absence of a clearly identified nuclease mediating the endonucleolytic cleavage, I believe that these data may be more appropriate for a specialist journal.

Other points:
- Figure 1e: To be convincing and demonstrate protection by ribosome/protein bound factors, Xrn1 treatment should be performed in extract and using RNA purified from the same extracts. Such data would directly provide evidence for protection. The same applies to Supplementary Figure 1b. Additional support for this model could eventually be provided by sucrose gradient analysis demonstrating that intermediates are found in the appropriate polysome fractions.
- Figure 1g and h: The loading control appears to be the same. Present the panels with a single loading control such that it is clear that this is the same membrane.
- Figure 3a: Why is species B1 not fully digested by Xrn1?
- Figure 5b: RNAs from the dom34 RGL1 strain should be presented.
- RNase I is sometime indicated as RNase I or RNase 1, please uniformize using the accepted nomenclature.

Please find below our response to Reviewers' comments :

Reviewer #1 (Remarks to the Author):

No-go decay (NGD) is a quality control mechanism essential for the degradation of aberrant transcript with propensity to cause ribosomal stall. It is well known that the mRNA is endonucleolytically cleaved near the stalled ribosome. However, precise mechanism that links ribosomal stall to the endonucleolytic cleavage has not been elucidated. In the present study, the authors suggest that (i) endonucleolytic cleavage occurs within the mRNA exit tunnel of the third or upstream ribosome numbered from the stalled site, and (ii) after removal of the third ribosome, the 5'-hydroxylated extremity of the 3'-NGD RNA fragment is phosphorylated by the Rlg1/Trl1 kinase, thereby enabling exonucleolytic degradation by the 5'-3' exonucleases Xrn1 and Dxo1. Overall, the data presented are generally convincing and interesting, however, a few issues still need to be resolved.

(1) All the results presented are based on the measure of the RNA fragments generated. From these results, the authors conclude that the cleavage of the RNA is due to the binding of the ribosome(s). There is no evidence showing that the RNA fragments (i.e., B1, B2, B3, B4, B5 RNA) observed in this study are resulting from the RNA protected by the binding of ribosome(s). The authors should show ribosome(s) actually bind to the RNA as disome, trisome, etc. by using polysome profile analysis. For example, B1 RNA mainly appeared in the dom34 background is fractionated to the disome?

> **Authors** : We performed polysome profile analyses, in parallel to in vitro RNAse protection assays that corroborate the association of the B1 and B5 RNAs with 2 or 3 ribosomes, respectively. The peak association of B4 is with 2 ribosomes, but with a significant portion remaining bound to 3 ribosomes. The polysome profiles are shown in Supplementary Fig. 1b, 1c , 1d and 1e.

(2) In Figure 2d and Supplementary Fig. 2d, the authors constructed three derivatives of mRNA1 named mRNA 2, 3 and 4. It is helpful for the reader to know the reason why the RNA sequences are designed as they are.

> **Authors** : The description of these derivatives of mRNA1 (mRNA 2, 3 and 4) has been removed from the revised manuscript for the sake of space.

(3) Figure 3a, Figure 5: the authors should provide direct evidence showing that B4 RNA has a hydroxyl group at its 5'-end (e.g., by using (i) phosphorylation of the 5'OH with T4PNK and 32P-ATP or (ii) oligonucleotide linker ligation followed by reverse transcription and PCR analysis (a method described by Peach et al., NAR 2015)).

> **Authors :** In addition to experiment shown in Fig. 3a, in this new version we provide more direct evidence that B4 RNA has a hydroxyl group at its 5'-end. These experiments were conducted by following the procedure described by A. Hopper (Wu et al., 2014) and are now shown in Fig. 5b and in Supplementary Fig. 5.

(4) In Figure 5a, B3 RNA is mainly observed in xrn1/dom34 cells, which appears to be inconsistent with the data in Figure 1-3. The authors should discuss it.

Authors : This is in fact an interesting observation about the activity of Dxo1 that we would like to study in the future. B2 and B3 RNA levels depend on Dxo1 activity as they disappear in a *dxo1* mutant strain (Fig. 2a). In the Figure 5a of our previous submitted manuscript, we observed that B2 RNAs are observed moreso than B3 RNAs. Interestingly, we observe that Dxo1 activity appears more important in the Yeast W303 genetic background than in the Yeast BY. This fluctuation of Dxo1 activity and its thermosensitivity are the reasons why we have inactivated Xrn1 and Dxo1 to study the importance of Rlg1 in the degradation of B4 RNAs. In this revised version, we provide more direct evidence of the role of Rlg1 by using a different approach (Fig. 5b and Supplementary Fig. 5). The description of the variable and thermosensitive activity of Dxo1 has been removed from the revised manuscript for the sake of space.

(5) Some of the nomenclatures used are very confusing. For example, the words “B4” and “Bd4” are used for the same 3’NGD RNA, and “RNA1” and “mRNA1” are used for 5’NGD RNA.

Authors: This has been corrected and clarified in the text.

(6) The manuscript contains a lot of typos/errors that need to be corrected throughout the manuscript.
Figure 1a legend: “Stop-less codon mRNA” should be “Stop codon-less mRNA”.
Figure 1b legend: “Agarose gel followed by”” should be “Agarose gel electrophoresis followed by”.
Figure 1b legend: “Probes prA, and prD” should be “Probes prA and prD”.
Figure 1h legend: “prA” should be “prD”.
Figure 2d legend: the word “RNA2, RNA3 and RNA4” is used in the legend, while “mRNA 2, 3, 4” is used in the main text, which is confusing to the reader.
Figure 4b legend: “prA” should be “prG” and “prG” should be “prA”.
Figure 5: “RGL1” should be “RLG1”.
Figure 6C does not appear in the main text.
Supplementary Fig. 5: “RGL1” should be “RLG1”.
Page 6, line 5 from the bottom: “hypothetised” should be “hypothesized”.
Page 11, line 5, “Supplementary Fig. 1e, 2b) should be “Fig. 1e and Supplementary Fig. 2b).
Page 13, line 4: the word “NGDase” must be explained here.
Page 15 line 7: “in vitro and in vitro” should be “in vitro and in vivo”.

Authors: All these typos/errors have been corrected.

Reviewer #2 (Remarks to the Author):

In this manuscript, Navickas and colleagues performed a detailed analysis of RNA fragments derived from No-Go decay reporters in mutant yeast strains and propose a

model for this process involving endonucleolytic cleavage of the target mRNA in the third ribosome blocked upstream of the stalling site (or ribosome present further 5').

While this study brings some new detailed information, it is unclear whether the proposed mechanism resulting from analysis of RNA decay intermediate accumulating in mutant cells, represents the main No-Go decay pathway. Indeed, most analyses are performed in mutants with inactivated Dom34. While based on bibliographic data authors argue that endonucleotlytic cleavage still occurs in this context, previous analyses have shown that full length mRNA stability is clearly increased in the absence of Dom34 indicating that endonucleolytic mRNA cleavage is blocked. The authors data presented in Figure 1b support further the fact that in the absence of Dom34 endonucleolytic RNA decay is strongly reduced (compare levels of full-length mRNA in the presence and absence of Dom34), leaving the possibility that RNA degradation events occurring in a dom34 mutant context represent minor side pathways. Consistently, previously published data suggested that the initial attack of No-Go decay substrate occurred much closer to the ribosome stall site.
Moreover, some of the data presented are difficult to reconcile with the authors' interpretation. Hence, authors propose that intermediates generated by endonucleolytic cleavage in the absence of Dom34 have a 5'OH group that is phosphorylated by Rgl1 before Xrn1 digestion. If this is indeed the case, it is unclear why the levels of B5 and B1 decay intermediates increase in a rgl1-4 dom34 double mutant compared to a single dom34 mutant (Figure 5a, compare to loading control). Indeed, according to the authors model, those B5 and B1 intermediates are expected to be dependant of Rgl1 activity and thus their levels are expected to decrease in the presence of a less active/inactive Rgl1.

Authors : We agree that it is crucial to show that these endonucleolytic cleavages occur in a *DOM34* background. For this purpose, we also performed 3' RACE experiments to confirm the existence of 5'NGD RNA in cells containing Dom34 (Fig. 3 and Fig. 6). Interestingly, the hel2 mutation was also shown to abolish the endonucleolytic cleavage (Ikeuchi et al., 2019). We verified that the production of the B4 RNA was completely abolished in a hel2 mutant, while the production of B1 and B5 RNAs was not affected (Supplementary Fig. 3a). This confirms that 5'-3' exoribonucleolytic degradation is also an important process in the production of NGD RNA fragments, independently of the endonucleolytic cleavage. This is also observed in Supplementary Fig. 6b and is in agreement with T. Inada's group and R. Green's group observations (Ikeuchi et al., 2019; D'orazio et al., 2019).

Overall, while this study presents a detailed study of decay intermediates derived from No-Go decay reporters in mutant yeast strains, the relation of these products to events occurring during No-Go decay in wild-type cells remain partly fuzzy. In the absence of a clearly identified nuclease mediating the endonucleolytic cleavage, I believe that these data may be more appropriate for a specialist journal.

Authors : We would like to mention that the bioRxiv version of this paper has already been cited by three important groups, Jay Hesselberth's, Rachel Green's and Joshua Arribere's respectively in (Cherry et al., 2019 eLife; D'Orazio et al., 2019 eLife

and Glover et al., 2019 in bioRxiv 2019 doi: https://doi.org/10.1101/674358). In particular, our finding that the RNA kinase activity of Rlg1 is important in the NGD pathway is particularly exciting for the RNA community, and provides important information for on-going studies on this NGD mRNA surveillance pathway.

Other points: Figure 1e: To be convincing and demonstrate protection by ribosome/protein bound factors, Xrn1 treatment should be performed in extract and using RNA purified from the same extracts. Such data would directly provide evidence for protection.

- **Authors** : Xrn1 treatment has been performed in cell extracts (in Fig. 1e) and using RNA purified from the same extracts in Fig. 3a, demonstrating the protection by ribosome/protein bound factors. We followed the same procedure to verify the protection by ribosome/protein bound factors upon RNase I treatment (Supplementary Fig. 1f and 1g).

The same applies to Supplementary Figure 1b. Additional support for this model could eventually be provided by sucrose gradient analysis demonstrating that intermediates are found in the appropriate polysome fractions.

Authors : We performed polysome profile analysis, in parallel to in vitro RNAse protection assays that corroborate the association of B1 and B5 RNAs with 2 or 3 ribosomes, respectively and the association of B4 with 2-3 ribosomes. Analyses of polysome profiles are shown in Supplementary Fig. 1b, 1c, 1d and 1e.

Figure 1g and h: The loading control appears to be the same. Present the panels with a single loading control such that it is clear that this is the same membrane.

- **Authors** : Reviewer is correct. It is the same membrane. We clarified this point in Fig. 1g.

Figure 3a: Why is species B1 not fully digested by Xrn1?

- **Authors** : we performed a limited digestion in time to show the high sensitivity of B4 RNA during Xrn1 treatment *in vitro.* In this new version, we also performed additional convincing experiments (Fig. 5b and in Supplementary Fig. 5) that clearly demonstrate the existence of 5'-hydroxyl B4 RNAs.

Figure 5b: RNAs from the dom34 RGL1 strain should be presented.

- **Authors** : We agree with the reviewer that it is important to analyse *RLG1* strain versus rlg1-4 mutant strain. By performing new experiments shown in Fig. 5b and in Supplementary Fig. 5, we now directly demonstrate that 5'-hydroxyl B4 RNAs accumulate in rgl1-4 mutant versus RlG1 strain.

RNase I is sometime indicated as RNase I or RNase 1, please uniformize using the accepted nomenclature.

<u>Authors:</u> This has been corrected. RNase I is now used in the text

Reviewers' comments:

Reviewer #1 (Remarks to the Author):

In this revised manuscript, the authors have performed additional experiments including (i) polysome profile analysis and (ii) phosphorylation status analysis of B4 RNA by taking reviewers' comments into account. The manuscript is improved but unfortunately has not in my opinion adequately addressed the concerns. Issues that remain are as follows:

(1) Polysome profile analysis in Supplementary Figure. 1b-1e

I do not find the polysome profiles to be convincing at all because of the following reasons:

First, the authors did not show the whole chart starting from the top free, 40S, 60S fractions. It appears that the polysome profiling was not appropriately performed, since the shape of the 80S peak looks odd.

Second, the fact that substantial amount of B1 RNA was detected in fraction 3~5+ where B1 RNA should not, further raising the concern that the experiment was not successfully performed. This point is particularly important because if RNAs are not well separated in the sucrose gradient or during fraction collection, it is hard to draw any conclusion regarding ribosome binding to specific RNAs. Again, I strongly recommend that RNA distribution should be examined in all the fraction from the top, including 40S and 60S, to the bottom to show in which fractions the peaks for B1, B4, B5 RNA species appear.

Lastly, the authors standardized the signal intensities using 5S rRNA to make a plot in Fig. S1e. This is terribly misleading, since there is no correlation between rRNA levels and the levels of the other types of RNAs in each fraction. The volume of each fraction should be the same, and plots should be indicated with the percent input.

Also, the authors described that "a portion of the B5 RNA was found to associate with three ribosomes. Although the major portion of the 71-nt B4 RNAs was associated with two ribosomes, a significant amount also associated with three ribosomes" (in p6 line 13-16). However, both B4 and B5 RNAs are mostly associated with two ribosomes based on the results in Supplementary Fig. 1b, d, and e.

(2) Phosporylation status analysis of B4 RNA in Figure 5 and Supplementary Figure 5

The data shown in these figures are not convincing. The effect of rlg1 ts mutant only marginally alters the phosphorylation status of B4 RNA. At 37°C, when compared to RLG1, both 5′-P and 5′-OH bands are similarly increased in rlg1 in both Fig5b and Fig. S5. The authors should perform time-course experiments to see if 5′-OH but not 5′-P form of B4 RNA is constantly accumulated. Also, it is recommended that the band intensities of 5′-P and 5′-OH forms are quantified, normalized and shown as a graph.

CIP treatment (in Fig. 5b lane 7) seems not to be performed appropriately. The amount of 5′-OH is not increased, in addition, that of both 5′-OH and 5′-P RNAs are decreased simultaneously (compare lane 5 and 7). The authors should reconfirm the experimental conditions of CIP treatment to be appropriate.

Although the authors use "higher resolution PAGE" to dissociate 5′-hydroxylated B4 RNAs from 5′-phosphorylated B4 RNAs (p11 line 27), the PAGE analysis is not mentioned in the METHODS section. Since Fig. 5 and Supplementary Fig. 5 is the most important data to conclude that Rlg1 phosphorylates No-go RNA product, the authors should describe it precisely in the METHODS section in

addition to referencing.

Reviewer #2 (Remarks to the Author):

With this revised manuscript, Navickas and colleagues argue of the importance of their findings for understanding NoGo Decay.
The strong points of this manuscript are:
- Detailed analyses of the decay intermediates accumulating in dom34 mutants;
- Identification of likely endonuclease cleavage sites related to NoGo Decay;
- Evidence supporting the presence of a 5'-OH at an RNA intermediate 5' end.

Authors have strengthened some conclusions of their manuscript in particular by demonstrating that formation of the B4 intermediate depends upon Hel2 and by better characterizing the B4 RNA intermediate 5' end.

Yet, some weaknesses are still present:
- Authors argue that the 5' end of the B4 intermediate is the main endonucleolytic cleavage during NoGo Decay. This is reflected in their model presented in Figure 7. Yet, the B5 intermediate appears to be more abundant than the B4 intermediate in a dom34 mutant. Given that B5 is longer than B4, the mechanism generating B5 remains unclear: B4 can't be a precursor of B5, if B4 is generated by endonucleolytic cleavage, how is B5 generated? Does this require decapping? Indeed, the scheme presented in Figure 7 fails to incorporate B5.
- It remains possible that the RNA degradation events occurring in a dom34 mutant context analyzed in this study represent minor side pathways of the NDG process.
- Accumulation of the B4 intermediate in the RLG1 mutant, and particularly of the 5'-OH ending species is not entirely convincing (Figure 5a and b, Supplementary Figure 5). These data presented are in sharp contrast with the black and white data presented by Wu and Hopper (Genes and Dev. (2014) 28:1556) using the same mutant.

Overall, while the authors have strengthened their manuscript, some conclusions remain weakly supported by the data shown. Furthermore, the manuscript remains confusing: the model presented doesn't explain all the data presented, conclusions regarding RLG1 are not entirely conclusive, the manuscript is very dense and contains data that are of limited value… Yet, this area is of high interest, especially in light of the recent publication on Cue2 and data submitted to bioRxiv. The current manuscript is probably more appropriate for a specialist journal, but Nature Communication could editorially decide to publish it as it reports new observations in a very competitive area. In the latter case, I believe that the manuscript should be extensively reformatted to more clearly present the strong conclusions, eliminate weak points, and facilitate its reading by a broad readership (suggestions below).

Detailed comments/suggestions:
- Authors should clarify the manuscript probably by stating that their data support the existence of multiple parallel pathways to degrade NGD substrates and that their observations suggest that one of these pathways still active if a dom34 mutant context involves endo cleavage of the mRNA in the 3rd paused ribosome. Indeed, there is no evidence that this is the main NGD pathway. The presence of the B5 intermediate demonstrates that other pathways, unexplained by the data presented here, are active. The description of different intermediates (e.g., by Doma and Parker) also suggest that multiple pathways are active. The model presented in Figure 7 should recapitulates all observation made in this manuscript (including production of B5, B3 and B2), with questions marks when necessary. Authors should emphasize the importance of detecting 5'-OH intermediates but limit the description of RLG1 as data supporting the latter conclusion are not entirely convincing. Authors also should remove superfluous/confusing data (e.g., impact of MET22…).

- In abstract and text, authors refer to the use of a ribozyme to generate 3’ truncated mRNA. This strategy having been pioneered by other groups, due credit should be given.
- Page 3: line 6 and 7: mRNA depurination is a chemical damage. The former could be deleted to shorten the manuscript.
- Page 8, line 20: B4 “is exclusively detected in Xrn1 deficient cells” contradicts results presented in Figure 1 showing that B4 is present in Dom34 XNR1+ cells. This should be clarified.
- Page 9, lines 6-14. This paragraph and the corresponding data (Figure 2c and Supplementary Figure 2c) bring little to this manuscript. This is just confusing for outsiders. This part should be deleted.
- Page 10, lines 11-12: describing 3’ RACE and calling it 3’ RACE is not helpful.
- Page 11, line 13-31: references are not correctly formatted for Nature Communications.
- Page 11, line 13-31: The usefulness of Figure 5a is unclear. Accumulation of the 5’-OH B4 intermediate in the rlg1 mutant is not convincing. Moreover, one would expect a decrease of the 5’-phosphorylated B4 species that is unclear. Altogether, the implication of RLG1 is not convincing, especially when the control data for that RNA intron are not as clear as the one presented in previous publications. The main finding is that a 5’OH species is present. Authors should focus their manuscript on this conclusion.
- Page 13, line 19: “that cleavage detected” is confusing because no cleavage is detected: An RNA species is detected… and probably doesn’t result from endonucleolytic cleavage.
- Page 14, lines 17-20: This seems to contradict the paragraph at the bottom of page 15.
- Authors use the symbol RLG1 for the yeast tRNA ligase. The official name being TRL1, this one may be more appropriate.
- Figure 3a: The incomplete digestion of the B1 species in the two right lanes should be explained.
- Figure 3b: What are the size of the two larger species?
- Figure 5: Accumulation of B4 in the rlg1-4 mutant is unclear, especially given the very unequal loading of the different lanes. Note further that the loading control signal is saturated and thus of little use.
- Page 27, line 10: “shifted” (not shift)
- Figure 7: How is B5 produced?
- Supplementary Figure 2b: Increase of the amount of the 47nt species at the expense of B4 is not convincing. Signal intensity appears quite variable. Have the authors quantified these data?

NCOMMS-18-34501B

We acknowledge that reviewers' suggestions have been very useful and improve our manuscript. Please note that to ensure the manuscript complies with Nature Communication editorial policies, we transferred for the sake of space two complete and unmodified paragraphs entitled "Ribosome protection of 3'-NGD RNA fragments from Xrn1 activity" and "Ribosome protection of 3'-NGD RNA fragments from RNase I activity" in section "Supplementary Information" (Pages 41 and 42). These paragraphs were selected because they didn't raise questions from reviewers.

Response to reviewers

Reviewers' comments:

<u>**Reviewer #1**</u> (Remarks to the Author):
In this revised manuscript, the authors have performed additional experiments including (i) polysome profile analysis and (ii) phosphorylation status analysis of B4 RNA by taking reviewers' comments into account. The manuscript is improved but unfortunately has not in my opinion adequately addressed the concerns. Issues that remain are as follows:
-Polysome profile analysis in Supplementary Figure. 1b-1e
I do not find the polysome profiles to be convincing at all because of the following reasons:
First, the authors did not show the whole chart starting from the top free, 40S, 60S fractions. It appears that the polysome profiling was not appropriately performed, since the shape of the 80S peak looks odd.
Second, the fact that substantial amount of B1 RNA was detected in fraction 3~5+ where B1 RNA should not, further raising the concern that the experiment was not successfully performed. This point is particularly important because if RNAs are not well separated in the sucrose gradient or during fraction collection, it is hard to draw any conclusion regarding ribosome binding to specific RNAs. Again, I strongly recommend that RNA distribution should be examined in all the fraction from the top, including 40S and 60S, to the bottom to show in which fractions the peaks for B1, B4, B5 RNA species appear.
Lastly, the authors standardized the signal intensities using 5S rRNA to make a plot in Fig. S1e. This is terribly misleading, since there is no correlation between rRNA levels and the levels of the other types of RNAs in each fraction. The volume of each fraction should be the same, and plots should be indicated with the percent input.
Also, the authors described that "a portion of the B5 RNA was found to associate with three ribosomes. Although the major portion of the 71-nt B4 RNAs was associated with two ribosomes, a significant amount also associated with three ribosomes" (in p6 line 13-16). However, both B4 and B5 RNAs are mostly associated with two ribosomes based on the results in Supplementary Fig. 1b, d, and e.
-<u>Authors</u>: Reviewer's suggestions were very useful. We performed and analysed polysome profiles as requested. These new data are shown in Fig. 1e and 1f and are discussed in page 6 lines 8 to 26. In addition, we mention in the Methods section that these experiments were performed in the absence of cycloheximide to prevent any drug-induced ribosome positioning, page 16 line 27.

<u>Reviewer #1</u>: Phosphorylation status analysis of B4 RNA in Figure 5 and Supplementary Figure 5
The data shown in these figures are not convincing. The effect of rlg1 ts mutant only marginally alters the phosphorylation status of B4 RNA. At 37°C, when compared to RLG1, both 5'-P and 5'-OH bands are similarly increased in rlg1 in both Fig5b and Fig. S5. The authors should perform time-course experiments to see if 5'-OH but not 5'-P form of B4 RNA is constantly accumulated. Also, it is recommended that the band intensities of 5'-P and 5'-OH forms are quantified, normalized and shown as a graph. CIP treatment (in Fig. 5b lane 7) seems not to be performed appropriately. The amount of 5'-OH is not increased, in addition, that of both 5'-OH and 5'-P RNAs are decreased simultaneously (compare lane 5 and 7). The authors should reconfirm the experimental conditions of CIP treatment to be appropriate.
-<u>Authors</u>: We agree that the use of the thermosensitive *rlg1-4* mutant may not be appropriate. To respond to reviewer's concerns, we used a viable *trl1Δ* mutant expressing pre-spliced intronless versions of the 10 intron-containing tRNAs (a generous gift from Jay Hesselberth). Using *trl1Δ* mutant, we observed a 24-fold accumulation of 5'-hydroxylated B4. We were also able to 5'-phosphorylate B4 RNA *in vitro* and confirm that this RNA can be digested by Xrn1 only after 5'-phosphorylation *in vitro*. We now conclude that the B4 RNA accumulates as a fully 5'-OH species in the *trl1Δ* mutant. These results are shown in Figure. 5a, 5b, 5c and 5d and Supplementary Fig. 5, and are discussed page 10 lines 3 to 17.

-<u>Reviewer #1</u>: Although the authors use "higher resolution PAGE" to dissociate 5'-hydroxylated B4 RNAs from 5'-phosphorylated B4 RNAs (p11 line 27), the PAGE analysis is not mentioned in the METHODS section. Since Fig. 5 and Supplementary Fig. 5 is the most important data to conclude that Rlg1 phosphorylates No-go RNA product, the authors should describe it precisely in the METHODS section in addition to referencing.
-<u>Authors</u>: We clarified this point by mentioning "RNAs were analyzed by 12% PAGE allowing separation of 5'-hydrolylated from 5'-phosphorylated RNAs", page 10 line 9-10, and also added a paragraph in the Methods section entitled "Gel electrophoresis for separation of RNA molecules", page 16 lines 10 to 13.

<u>Reviewer #2</u>: (Remarks to the Author):
With this revised manuscript, Navickas and colleagues argue of the importance of their findings for understanding NoGo Decay.
The strong points of this manuscript are:
- Detailed analyses of the decay intermediates accumulating in dom34 mutants;
- Identification of likely endonuclease cleavage sites related to NoGo Decay;
- Evidence supporting the presence of a 5'-OH at an RNA intermediate 5' end.

Authors have strengthened some conclusions of their manuscript in particular by demonstrating that formation of the B4 intermediate depends upon Hel2 and by better characterizing the B4 RNA intermediate 5' end.
Yet, some weaknesses are still present:
Authors argue that the 5' end of the B4 intermediate is the main endonucleolytic cleavage during NoGo Decay. This is reflected in their model presented in Figure 7. Yet, the B5 intermediate appears to be more abundant than the B4 intermediate in a dom34 mutant. Given that B5 is longer than B4, the mechanism generating B5 remains unclear: B4 can't be a

precursor of B5, if B4 is generated by endonucleolytic cleavage, how is B5 generated? Does this require decapping? Indeed, the scheme presented in Figure 7 fails to incorporate B5.

-<u>Authors:</u> We agree and we take into account the existence of alternative pathways. We modified the Figure 7 and its legend. We also discussed this model in page 14, lines 19 to 33 and page 15, lines 1 to 10.

-<u>Reviewer #2</u>: It remains possible that the RNA degradation events occurring in a dom34 mutant context analyzed in this study represent minor side pathways of the NDG process.

Accumulation of the B4 intermediate in the RLG1 mutant, and particularly of the 5'-OH ending species is not entirely convincing (Figure 5a and b, Supplementary Figure 5). These data presented are in sharp contrast with the black and white data presented by Wu and Hopper (Genes and Dev. (2014) 28:1556) using the same mutant.

-<u>Authors:</u> In the light of our new results using *trl1Δ* mutant, we hope that we respond to reviewer's concerns (also see response to reviewer 1 above). Results are shown in Figure. 5a, 5b, 5c and 5d and Supplementary Fig. 5, and are discussed in page 10 lines 3 to 17.

-<u>Reviewer #2</u>: Overall, while the authors have strengthened their manuscript, some conclusions remain weakly supported by the data shown. Furthermore, the manuscript remains confusing: the model presented doesn't explain all the data presented, conclusions regarding RLG1 are not entirely conclusive, the manuscript is very dense and contains data that are of limited value… Yet, this area is of high interest, especially in light of the recent publication on Cue2 and data submitted to bioRxiv. The current manuscript is probably more appropriate for a specialist journal, but Nature Communication could editorially decide to publish it as it reports new observations in a very competitive area. In the latter case, I believe that the manuscript should be extensively reformatted to more clearly present the strong conclusions, eliminate weak points, and facilitate its reading by a broad readership (suggestions below).

Detailed comments/suggestions:

Authors should clarify the manuscript probably by stating that their data support the existence of multiple parallel pathways to degrade NGD substrates and that their observations suggest that one of these pathways still active if a dom34 mutant context involves endo cleavage of the mRNA in the 3rd paused ribosome. Indeed, there is no evidence that this is the main NGD pathway. The presence of the B5 intermediate demonstrates that other pathways, unexplained by the data presented here, are active. The description of different intermediates (e.g., by Doma and Parker) also suggest that multiple pathways are active. The model presented in Figure 7 should recapitulates all observation made in this manuscript (including production of B5, B3 and B2), with questions marks when necessary. Authors should emphasize the importance of detecting 5'-OH intermediates but limit the description of RLG1 as data supporting the latter conclusion are not entirely convincing.

-<u>Authors:</u> We modified the Figure 7 that now recapitulates all observations made in this manuscript. In the light of our new results using the *trl1Δ* mutant, we hope that reviewer will agree that our data now supports our main conclusion.

-<u>Reviewer #2</u>: Authors also should remove superfluous/confusing data (e.g., impact of MET22…).

-**<u>Authors:</u>** We agree and removed this part.

Reviewer #2: In abstract and text, authors refer to the use of a ribozyme to generate 3' truncated mRNA. This strategy having been pioneered by other groups, due credit should be given.
-Authors: We cite a publication from Ambro van Hoof's group. Page 4 line 1.

-Reviewer #2:-Page 3: line 6 and 7: mRNA depurination is a chemical damage. The former could be deleted to shorten the manuscript.
-Authors: This has been corrected.

-Reviewer #2: Page 8, line 20: B4 "is exclusively detected in Xrn1 deficient cells" contradicts results presented in Figure 1 showing that B4 is present in Dom34 XNR1+ cells. This should be clarified.
-Authors: We agree that this is confusing, it was a mistake, the sentence has now been corrected (page 7 lines 4 to 6): "We strongly suspected that the B4 species was the original NGD product, and because B3 and B2 RNAs were exclusively detected in Xrn1 deficient cells, we speculated that these RNAs might be derived from B4 by an alternative 5'-3' exoribonuclease."

-Reviewer #2: Page 9, lines 6-14. This paragraph and the corresponding data (Figure 2c and Supplementary Figure 2c) bring little to this manuscript. This is just confusing for outsiders. This part should be deleted.
-Authors: We agree, this has been deleted.

-Reviewer #2: Page 10, lines 11-12: describing 3' RACE and calling it 3' RACE is not helpful.
-Authors: This has been simplified.

-Reviewer #2: Page 11, line 13-31: references are not correctly formatted for Nature Communications.
-**Authors:** This has been corrected.

-Reviewer #2: Page 11, line 13-31: The usefulness of Figure 5a is unclear. Accumulation of the 5'-OH B4 intermediate in the rlg1 mutant is not convincing. Moreover, one would expect a decrease of the 5'-phosphorylated B4 species that is unclear. Altogether, the implication of RLG1 is not convincing, especially when the control data for that RNA intron are not as clear as the one presented in previous publications. The main finding is that a 5'OH species is present. Authors should focus their manuscript on this conclusion.
-Authors: In the light of our new results using the *trl1Δ* mutant, we hope that reviewer will agree that our data now supports our conclusion. Results are shown in Figure 5a, 5b, 5c, 5d and Supplementary Fig. 5, and are discussed in page 10 lines 3 to 17.

-**Reviewer #2:** Page 13, line 19: "that cleavage detected" is confusing because no cleavage is detected: An RNA species is detected… and probably doesn't result from endonucleolytic cleavage.
-**Authors:** We agree that this was confusing. This has been modified to "We demonstrate that primer extension arrests detected in the region covered by disomes are abolished in *dxo1*/*xrn1* mutant cells, suggesting that they are the products of subsequent trimming by these enzymes", page 12 line lines 6 to 8.

-**Reviewer #2:** Page 14, lines 17-20: This seems to contradict the paragraph at the bottom of page 15.
-**Authors**: This sentence was confusing, and it has been corrected (page 12 lines 29 to 33): "This localizes the 5'-extremity of cleaved RNA within the mRNA exit tunnel, 4 nts downstream of the expected nucleotide position of a canonical mRNA that emerges from the ribosome and becomes available for cleavage by RNase I *in vitro*, classically used in ribosome foot-printing studies".

-**Reviewer #2:** Authors use the symbol RLG1 for the yeast tRNA ligase. The official name being TRL1, this one may be more appropriate.
-**Authors:** We agree, and this has been corrected. We use now TRL1.

-**Reviewer #2:** Figure 3a: The incomplete digestion of the B1 species in the two right lanes should be explained.
-**Authors:** Xrn1 digestion in kinase buffer is not always optimal. This is explained in page 8 lines 6 to 9: "A portion of the abundant B1 RNAs persisted during Xrn1 treatment in kinase buffer (Fig. 3a, and see Methods), but parallel Xrn1 digestion in optimal buffer confirms that B1 and B5 RNAs were totally digested (*i.e*., are fully mono-phosphorylated), while the B4 RNA remained resistant (Supplementary Fig. 3a)".

-**Reviewer #2:** Figure 3b: What are the size of the two larger species?
-**Authors:** Reviewer talks certainly about the two larger species visible in Figure 3c. These two larger species have a size of above 150bp. We did not extract amplicons larger than 150bp because such PCR products do not correspond to any potentially cleaved mRNA1. This is discussed in page 13 lines 11 to 16: "Our 3'-RACE experiments did not amplify DNA products corresponding to RNAs corresponding to the predicted sizes of NGD-cleaved RNAs with the second (41 nts) or first stalled ribosome (15 nts) (predicted sizes 95 and 125 nts, respectively), indicating that they do not occur to any significant level. The major ~65-bp RT-PCR products corresponded perfectly to RNAs cleaved 71nt upstream of the 3'-extremity of mRNA1, suggesting this is the primary site of NGD cleavage."

-**Reviewer #2:** Figure 5: Accumulation of B4 in the rlg1-4 mutant is unclear, especially given the very unequal loading of the different lanes. Note further that the loading control signal is saturated and thus of little use.
-Authors: New results using the *trl1Δ* strain are now shown in Figure 5a, 5b, 5c, 5d and Supplementary Fig. 5, and are discussed in page 10 lines 3 to 17.

-**Reviewer #2:** Page 27, line 10: "shifted" (not shift)
-**Authors:** This has been corrected.

-**<u>Reviewer #2:</u>** Figure 7: How is B5 produced?
-**<u>Authors:</u>** We modified Figure 7 and its legend. We discussed this model and the production of B5 in page 14, lines 19 to 33 and page 15, lines 1 to 10.

-**<u>Reviewer #2:</u>** Supplementary Figure 2b: Increase of the amount of the 47nt species at the expense of B4 is not convincing. Signal intensity appears quite variable. Have the authors quantified these data?
-**<u>Authors:</u>** We modified this sentence, page 7 lines 19 to 21: "the decrease in the amount of B4 RNA was correlated with an almost equivalent increase of a 47-nt species suggesting that disomes persist on the majority of the 3'-ends of B4 RNAs in *dom34/xrn1/dxo1* cells *in vivo*" and this has also been quantified using two independent experiments, quantification is shown in Supplementary Fig. 2c."

Reviewers' comments:

Reviewer #1 (Remarks to the Author):

The authors have responded fully to each of the points raised in my previous review of the manuscript. They have made a strong study even more convincing. Therefore, I am happy to recommend the revised manuscript for publication.

Reviewer #2 (Remarks to the Author):

This revised manuscript by Benard and co-workers has been modified to answer previous referees' comments. It is particularly strengthened by the replacement of experiments using a conditional mutant of the tRNA ligase for a totally inactivated allele. Evidence that some RNA fragments derived from a NoGo substrate carry a 5' hydroxyl is now really convincing. The authors also clarified the manuscript that is easier to read and added a summary figure (Figure 7) that should help anyone to understand the model that they propose and discuss.

Yet, I believe that 2 points raise questions:

1) The authors argue (page 7, line 5 from bottom) RNA species B4 "is the major 3'-product of NGD cleavage" in their construct. However, indicates RNA species B4 is present at levels that are lower than RNA species B5 that is, like B4, only observed in a dom34 mutant (Figure 1c lane 2 and Figure 1e). This argues that B4 is not the major 3'-product of NGD cleavage as upstream cleavage(s) is(are) likely to be more frequent. The data presented don't exclude that the pathway leading to endonucleolytic cleavage at B4 is a minor NGD pathway.

2) I should have noticed earlier that the data presented to argue that the two fragments originating from an endonucleolytic cleavage at B4 have been identified don't support this conclusion! I apologize for not bringing this earlier.
Indeed, in their model the authors propose that cleavage at B4 generates a 5'-OH group, thus the 3' end of the upstream species should have a 3' phosphate (or2'-3' cyclic phosphate). In either case, it wouldn't be possible to ligate a downstream primer. Yet, the strategy used by the authors to identify the upstream product involve the ligation of such a downstream primer (Figure 3)! This is a serious weakness. The data presented thus are not as strong as the authors indicate to support an endonucleolytic cleavage generating a 5'-OH extremity. While the latter remains possible, an alternative, as likely, possibility is that an endonucleolytic event generates a 3'-OH and 5'-phosphate but that the later extremity gets dephosphorylated.

Altogether, even if the authors have improved their manuscript with the use of the Trl1 deletion mutant, several weaknesses and issues remain. Those would need to be addressed before publication.

NCOMMS-18-34501C

Response to reviewers

Reviewer #1 (Remarks to the Author):

The authors have responded fully to each of the points raised in my previous review of the manuscript. They have made a strong study even more convincing. Therefore, I am happy to recommend the revised manuscript for publication.

Reviewer #2 (Remarks to the Author):

This revised manuscript by Benard and co-workers has been modified to answer previous referees' comments. It is particularly strengthened by the replacement of experiments using a conditional mutant of the tRNA ligase for a totally inactivated allele. Evidence that some RNA fragments derived from a NoGo substrate carry a 5' hydroxyl is now really convincing. The authors also clarified the manuscript that is easier to read and added a summary figure (Figure 7) that should help anyone to understand the model that they propose and discuss.

Yet, I believe that 2 points raise questions:

1) The authors argue (page 7, line 5 from bottom) RNA species B4 "is the major 3'-product of NGD cleavage" in their construct. However, indicates RNA species B4 is present at levels that are lower than RNA species B5 that is, like B4, only observed in a dom34 mutant (Figure 1c lane 2 and Figure 1e). This argues that B4 is not the major 3'-product of NGD cleavage as upstream cleavage(s) is(are) likely to be more frequent. The data presented don't exclude that the pathway leading to endonucleolytic cleavage at B4 is a minor NGD pathway.

Authors: Fig.1 is not the best figure to get an idea of the importance of the B4 cleavage relative to B1 and B5. Its relative importance only becomes clear when degradation is blocked in *xrn1/dxo1* mutant (Figures 2a and 2b) or *trl1* mutant (Figures 5a and 5b). In the former, B4 is the major species observed, and in the latter it is at least two-fold more abundant than the generated B1 RNAs and clearly more abundant than B5 RNAs. We nonetheless attenuated the sentence in question (lines 30-31 page 7): "The results described above suggest that the principal band detected in the absence of Xrn1 and Dxo1 (B4 RNA) is a specific 3'-product of NGD cleavage in our constructs (Fig. 2a)." Figure 7 takes into account the existence of alternative degradation pathways in which we have taken care not to present B4 RNAs as major products of NGD.

2) I should have noticed earlier that the data presented to argue that the two fragments originating from an endonucleolytic cleavage at B4 have been identified don't support this conclusion! I apologize for not bringing this earlier.

Indeed, in their model the authors propose that cleavage at B4 generates a 5'-OH group, thus the 3' end of the upstream species should have a 3' phosphate (or 2'-3' cyclic phosphate). In either case, it wouldn't be possible to ligate a downstream primer. Yet, the strategy used by the authors to identify the upstream product involve the ligation of such a downstream primer (Figure 3)! This is a serious weakness. The data presented thus are not as strong as the authors indicate to support an endonucleolytic cleavage generating a 5'-OH extremity. While the latter remains possible, an alternative, as likely, possibility is that an endonucleolytic event generates a 3'-OH and 5'-phosphate but that the later extremity gets dephosphorylated.

Altogether, even if the authors have improved their manuscript with the use of the Trl1 deletion mutant, several weaknesses and issues remain. Those would need to be addressed before publication.

Authors: "RNAs were pre-treated with T4 polynucleotide kinase to modify 2'-3' cyclic phosphates to 3'-OH to permit RNA ligation[31]". This sentence was added on page 8, lines 20-22 to better clarify the procedure for the detection of these RNA species. This information about the modified 3'-RACE procedure was present in the Methods section and in the text of previous versions of the manuscript, but was removed from the text in the latest version to simplify, at reviewer 2's request. Although we haven't formally ruled it out, we consider that the alternative 3'OH/5'P scenario proposed by reviewer 2, is less likely than direct production of a 5'-OH, since it would involve removing a 5'-phosphate by a hypothetical 5'-RNA phosphatase (not known to exist in yeast), only to restore it again by Trl1.
